# Tick-borne bacterial pathogens in southwestern Finland

**DOI:** 10.1186/s13071-016-1449-x

**Published:** 2016-03-22

**Authors:** Jani J. Sormunen, Ritva Penttinen, Tero Klemola, Jari Hänninen, Ilppo Vuorinen, Maija Laaksonen, Ilari E. Sääksjärvi, Kai Ruohomäki, Eero J. Vesterinen

**Affiliations:** Department of Biology, University of Turku, FI-20014 Turku, Finland; Archipelago Research Institute, University of Turku, FI-20014 Turku, Finland; Zoological Museum, Department of Biology, University of Turku, FI-20014 Turku, Finland; Department of Agricultural Sciences, University of Helsinki, FI-00014 Helsinki, Finland

**Keywords:** *Ixodes ricinus*, *Ixodes persulcatus*, Tick-borne diseases, *Borrelia burgdorferi*, *Borrelia miyamotoi*, *Rickettsia*, *Bartonella*, *Candidatus* Neoehrlichia mikurensis, Finland

## Abstract

**Background:**

*Ixodes ricinus* and *Ixodes persulcatus* are the main vectors of Lyme borreliosis spirochetes and several other zoonotic bacteria in northern Europe and Russia. However, few studies screening bacterial pathogens in Finnish ticks have been conducted. Therefore, reports on the occurrence and prevalence of several bacterial pathogens detected from ticks elsewhere in Europe and Russia are altogether missing from Finland. The main aim of the current study was to produce novel data on the occurrence and prevalence of several tick-borne bacterial pathogens in ticks collected from southwestern Finland.

**Methods:**

Ticks were collected in 2013–2014 by blanket dragging from 25 localities around southwestern Finland, and additionally from a dog in Lempäälä. Collected ticks were molecularly identified and screened for *Borrelia burgdorferi* s.l., *Borrelia miyamotoi, Rickettsia*, *Bartonella* and *Candidatus* Neoehrlichia mikurensis using quantitative PCR. Furthermore, detected *Rickettsia* spp. were sequenced using conventional PCR to determine species.

**Results:**

A total of 3169 ticks in 1174 DNA samples were screened for the listed pathogens. The most common bacteria detected was *B. burgdorferi* (*s.l*.) (18.5 % nymphal and 23.5 % adult ticks), followed by *Rickettsia* spp. (1.1 %; 5.1 %) and *B. miyamotoi* (0.51 %; 1.02 %). *B. miyamotoi* and *Rickettsia* spp. were also detected in larval samples (minimum infection rates 0.31 % and 0.21 %, respectively). Detected *Rickettsia* spp. were identified by sequencing as *R. helvetica* and *R. monacensis*. All screened samples were negative for *Bartonella* spp. and *Ca.* N. mikurensis.

**Conclusions:**

In the current study we report for the first time the presence of *Rickettsia* in Finnish ticks. Furthermore, *Rickettsia* spp. and *B. miyamotoi* were found from larval tick samples, emphasizing the importance they may have as vectors of these pathogens. Comparisons of tick density estimates and *B. burgdorferi* (*s.l*.) prevalence made between the current study and a previous study conducted in 2000 in ten out of the 25 study localities suggest that an increase in tick abundance and *B. burgdorferi* (*s.l*.) prevalence has occurred in at least some of the study localities.

## Background

Ticks (Acari: Ixodida) are the primary vectors for several severe zoonotic infections worldwide [1, 2]. A recent survey of 335 emerging infectious disease events indicated that 60.3 % of emerging diseases are zoonoses and that the amount of zoonoses is increasing over time [3]. In Northern Europe and Western Russia, Lyme borreliosis caused by *Borrelia burgdorferi* (*sensu lato*) spirochetes is among the most common and severe zoonotic infections frequently affecting humans [4]. The primary vectors for *B. burgdorferi* (*s.l*.) are hard ticks (Ixodidae), in northern Europe and Russia specifically *Ixodes ricinus* (Linnaeus, 1758) and *Ixodes persulcatus* (Schulze, 1930). However, in addition to *B. burgdorferi* (*s.l*.), humans bitten by ixodids may also be infected with pathogens from several other bacterial genera, which can present themselves through various clinical manifestations.

Bacteria of the genus *Borrelia* are commonly found in ticks all over the world. These spirochetes are the causative agents for two well-defined human infections: Lyme borreliosis and relapsing fever. Lyme borreliosis is caused by *Borrelia* spp. belonging to the *B. burgdorferi* (*s.l.*) species complex. Several *B. burgdorferi* (*s.l*.). genospecies have been reported from *I. persulcatus* and *I. ricinus* in Europe, most of which have been shown to cause Lyme borreliosis [5] and have known reservoir hosts [6]. More recently, a species of *Borrelia* not belonging to the *B. burgdorferi* (*s.l*.) species complex has been reported from hard ticks in Europe. This species, *Borrelia miyamotoi*, belongs to the relapsing fever group of *Borrelia*, which are usually transmitted by soft ticks (Argasidae). Following the initial detection and description of the pathogen from hard ticks (*Ixodes ovatus*) in Japan [7], reports of *B. miyamotoi* or *B. miyamotoi-*like bacteria in *Ixodes* ticks have been published from over a dozen European countries [8–10]. Whereas Lyme borreliosis cases are relatively common and well documented [4], human patient cases linked with *B. miyamotoi* infection have only been reported recently [11–15].

The bacterial genus *Rickettsia* is traditionally classified into two groups: the spotted fever group (SFG) and the typhus group [16]. Species from both groups have been found from hard ticks. In Europe, particularly SFG *Rickettsia* are frequently reported from questing ticks [17–21], feeding ticks removed from host animals [22–24], and tissue of host animals [25]. In Northern Europe, *Rickettsia helvetica* have been reported from *Ixodes* ticks in Sweden [26–29], Denmark [30, 31], Estonia [32], and Lithuania and Latvia [33]. Furthermore, *R. monacensis* and *Candidatus* R. tarasevichiae have been reported from Estonian tick populations [32]. Patient cases from Spain and China suggest that the latter two *Rickettsia* spp. are capable of human infection [34, 35].

*Bartonella* is a genus of gram-negative intracellular bacteria, several species of which are considered to have the potential for human infection [36]. The role of *I. ricinus* as vectors for *Bartonella* spp. has recently begun to receive increasing scientific attention [36–42]. However, while DNA of various *Bartonella* spp. has been found from ticks [36], actual vector competence of ticks has only been shown for a few [39, 43].

*Candidatus* Neoehrlichia mikurensis is a candidate-status species of gram-negative cocci belonging to the family Anaplasmataceae. The first case of human infection in Europe was reported from Sweden, in a patient with recurrent fever episodes [44]. This was soon followed by reports of patient cases in Switzerland and Germany [45, 46]. *Candidatus* N. mikurensis has been reported from rodents and ticks in Europe, though actual vector competence of ticks has not been shown [47–53]. Nevertheless, due to the high prevalence of the pathogen observed in ticks and rodents in some of the cited studies, it should be considered as a potential tick-borne pathogen.

Few studies focusing on tick-borne bacterial pathogens have been conducted in Finland. Consequently, reports regarding the occurrence and prevalence of many tick-borne bacterial pathogens found from ticks elsewhere in Europe do not exist for Finland. Concerning *Borrelia, Rickettsia, Bartonella* and *Ca.* N. mikurensis, published data of prevalence in Finnish ticks exists only for *B. burgdorferi* (*s.l*.) and *B. miyamotoi* [9, 10, 54–57]. The main aim of the current study was to produce novel data of tick-borne bacterial pathogens in Finnish ticks by determining the occurrence and prevalence of *Borrelia burgdorferi* (*s.l.*), *B. miyamotoi, Rickettsia*, *Bartonella* and *Candidatus* Neoehrlichia mikurensis in ticks collected from southwestern Finland.

## Methods

### Tick collection and sample preparation

**Fig. 1:**
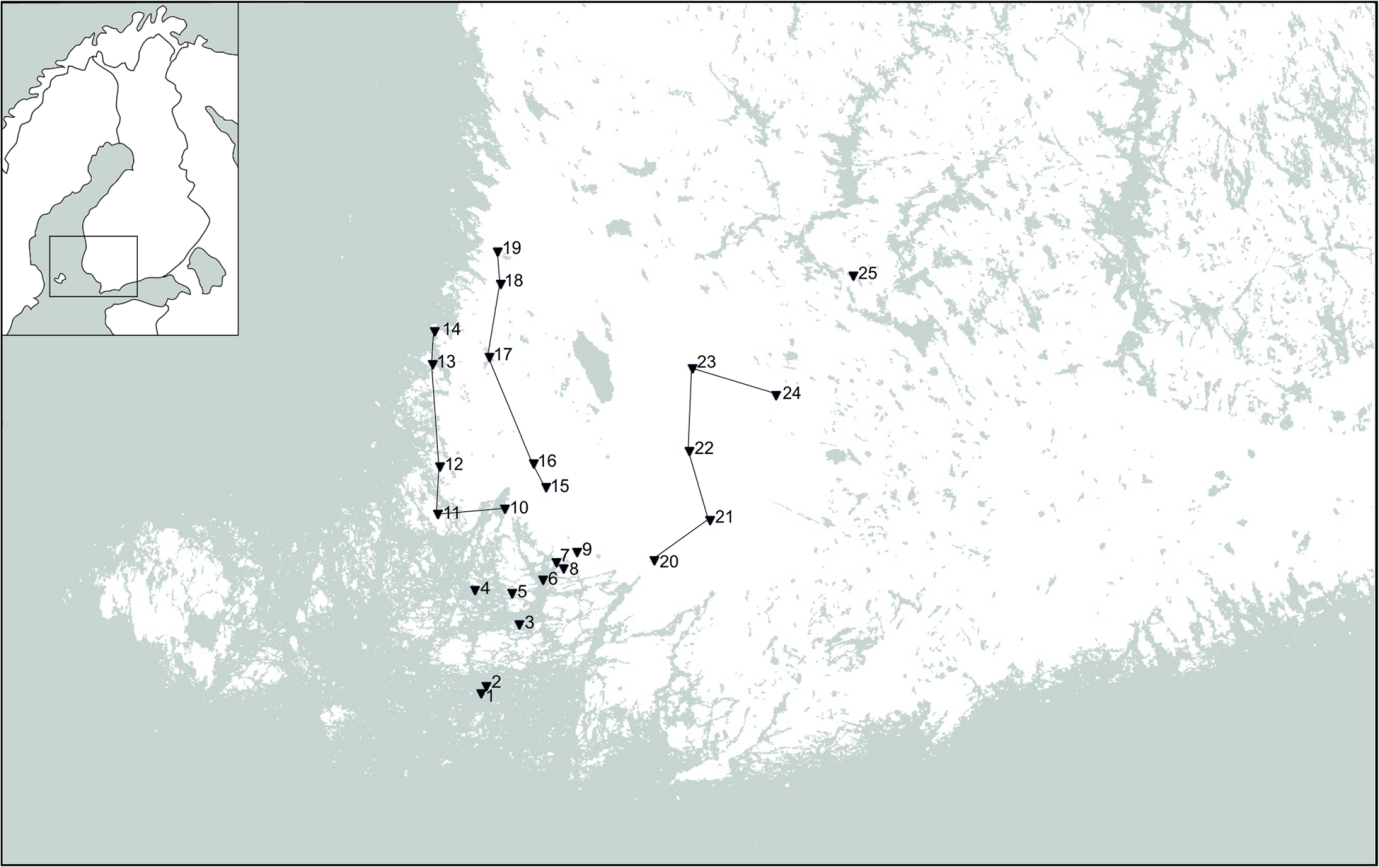
Field survey and dog tick collection localities in southwestern Finland. 1. Boskär 2. Berghamn 3. Seili 4. Pähkinäinen 5. Maisaari 6. Vepsä 7. Ruissalo 8. Hirvensalo 9. City of Turku; city parks Samppalinna and Urheilupuisto 10. Askainen 11. Kustavi 12. Lokalahti 13. Pyhämaa 14. Pyhäranta 15. Nousiainen 16. Laitila 17. Kodisjoki 18. Eurajoki 19. Luvia 20. Paimio 21. Marttila 22. Mellilä 23. Matku 24. Alastaro 25. Lempäälä

Ticks were collected from May (week 20) to September (week 39) in 2013 and 2014 from 25 localities around southwestern Finland (Fig. 1). Ticks were sampled by slowly dragging a 1.0 m^2^ square cotton cloth along ground floor vegetation in 10 m sections, for a total of 50–200 m per dragging session in each locality, apart from one of the islands, Seili, where 750 m were dragged per session. Sampling was done every other or every third week, depending on locality. In Seili, dragging was conducted on study transects as previously described in Sormunen *et al*. [10]. Elsewhere, dragging was conducted in coniferous and deciduous forests and alder thickets, following classification from a previous study [10]. Furthermore, ticks were collected from a domestic dog in Lempäälä, the only known *I. persulcatus* focus in southwestern Finland (Fig. 1). Despite recurring tick infestations, to our knowledge, the dog in question has never suffered from any tick-borne diseases.

Ten out of the 25 field survey localities were previously studied by Mäkinen *et al.* [54]: two city parks in Turku (Samppalinna and Urheilupuisto), two suburban islands (Hirvensalo and Ruissalo) and six rural islands in the Turku archipelago (Seili, Böskar, Berghamn, Pähkinäinen, Maisaari, Vepsä). The remaining 15 study localities had not been investigated before. These novel localities were grouped into three major study lines for logistical reasons: the Coastal line (Askainen, Kustavi, Lokalahti, Pyhämaa, Pyhäranta), the Northern line (Nousiainen, Laitila, Kodisjoki, Eurajoki, Luvia), and the Northwestern line (Alastaro, Matku, Mellillä, Marttila, Paimio) (Fig. 1).

All ticks attached to the cloth were collected using tweezers after each 10 m drag. Larvae, nymphs, and adults were separated and placed in 1.5 ml Eppendorf tubes. Each adult and nymph was placed in its own tube, with the exception of 153 nymphs, which were placed in 37 randomly sized pools. All larvae collected during a single dragging session (5–20 drags, 50–200 m) in each locality were placed in a single tube. The ticks were then transported alive to the Department of Biology in the University of Turku for deep freezing (-80 °C).

Total DNA was extracted from frozen tick samples between June 2014 – March 2015 using NucleoSpin® RNA kits and RNA/DNA buffer sets (Macherey-Nagel, Germany), following the kit protocols (RNA Kit: Rev. 16/May 2014 and RNA/DNA buffer set: Rev. 08/May 2014). RNA extracts were stored at -80 °C for later use. DNA extracts were stored at -20 °C.

Analyses regarding tick species and *Borrelia* spp. prevalence were carried out on individual DNA samples. For screening of *Rickettsia*, *Bartonella*, and *Candidatus* Neoehrlichia mikurensis, aliquots of original DNA samples were pooled (ten samples per pool, 5 μl of each sample) to make the procedure faster and more cost-efficient due to low expected prevalence. Original, separate DNA samples were re-analyzed as needed when a pooled sample was found positive.

### Real-time qPCR

Real-time quantitative PCR (henceforth abbreviated qPCR) assays were carried out using KAPAProbe FAST PCR Kit (product number KK4706; KAPA Biosystems, Wilmington, Massachusetts, USA). All samples were analyzed in three replicate reactions carried out on 384-well plates. At least three blank water samples were used as negative controls in each assay. The thermal cycling profile used was 95 °C for 3 min, then 50 cycles of 95 °C for 3 s and 60 °C for 30 s. Thermal cycling was carried out at Finnish Microarray and Sequencing Centre (FMSC, Turku, Finland) using QuantStudio 12 K Flex Real-Time PCR System (Life Technologies Inc. [LTI], Carlsbad, CA). All qPCR results were analyzed using QuantStudio™ 12 K Flex Software v.1.2.2 (LT1). Samples were considered positive only when successful amplification was detected in all three replicate reactions.

### Tick species determination

Tick species was determined in a species-specific duplex qPCR assay. We designed primers IXO-I2-F4 and IXO-I2-R4 targeting the *Ixodes* spp. *ITS2* gene to amplify genus specific segments (Table 1). Species-specific probes Ipe-I2-P4 and Iri-I2-P4 were designed to match either of the tick species (*I. persulcatus* and *I. ricinus*, respectively) (Table 1). This approach is very cost-efficient and gives the advantage to simultaneously detect both species present in large pools. Assays were carried out in 5 μl reaction volume, including 2.5 μl KAPAProbe FAST PCR Kit, 200 nM forward primer, 200 nM reverse primer, 150 nM Iri-I2-P4 probe, 100 nM Ipe-I2-P4 probe, 1.175 μl ddH2O, and 1 μl DNA. The newly designed primers and probes were tested by amplifying hundreds of sequenced *I. ricinus* and *I. persulcatus* DNA samples from an earlier study [10]. DNA samples of *I. ricinus* and *I. persulcatus* confirmed by sequencing were used as positive controls in each assay.

**Table 1:** Primers and probes used in tick species determination and pathogen screening

Primer/probe name	Primer/probe target	5’ → 3’	Reference
qPCR:			
IXO-I2-F4	*Ixodes* spp. *ITS2*	TCTCGTGGCGTTGATTTGC	This paper
IXO-I2-R4	*Ixodes* spp. *ITS2*	CTGACGGAAGGCTACGACG	
Ipe-I2-P4	*I. persulcatus ITS2*	[FAM]-TGCGTGGAAAGAAAACGAG-[BHQ1]	
Iri-I2-P4	*I. ricinus ITS2*	[VIC]-TGCTCGAAGGAGAGAACGA-[BHQ1]	
Bb23Sf	*B. burgdorferi* 23S RNA	CGAGTCTTAAAAGGGCGATTTAGT	Courtney et al. [83]
Bb23Sr	*B. burgdorferi* 23S RNA	GCTTCAGCCTGGCCATAAATAG	
Bb23Sp	*B. burgdorferi* 23S RNA	[FAM]-AGATGTGGTAGACCCGAAGCCGAGTG-[BHQ1]	
Bmi-F	*B. miyamotoi glpQ*	CACGACCCAGAAATTGACACA	Vayssier-Taussat et al. [84]
Bmi-R	*B. miyamotoi glpQ*	GTGTGAAGTCAGTGGCGTAAT	
Bmi-P	*B. miyamotoi glpQ*	[FAM]-TCGTCCGTTTTCTCTAGCTCGATTGGG-[BHQ1]	
Bart-ssRA-F	*Bartonella ssRa*	GCTATGGTAATAAATGGACAATGAAATAA	Diaz et al. [85]
Bart-ssRA-R	*Bartonella ssRa*	GCTTCTGTTGCCAGGTG	
Bart-ssRA-P	*Bartonella ssRa*	[FAM]-ACCCCGCTTAAACCTGCGACG-[BHQ1]	
Rspp-F	*Rickettsia gltA*	GAGAGAAAATTATATCCAAATGTTGAT	Labruna et al. [86]
Rspp-R	*Rickettsia gltA*	AGGGTCTTCGTGCATTTCTT	
Rspp-P	*Rickettsia gltA*	[CY5]-CATTGTGCCATCCAGCCTACGGT-[BHQ3]	
CNe-F	*Ca.* N. mikurensis *groEL*	AGAGACATCATTCGCATTTTGGA	Vayssier-Taussat et al. [84]
CNe-R	*Ca.* N. mikurensis *groEL*	TTCCGGTGTACCATAAGGCTT	
CNe-P	*Ca.* N. mikurensis *groEL*	[TAMRA]-AGATGCTGTTGGATGTACTGCTGGACC-[BHQ2]	
PCR:			
CS877f	*Rickettsia gltA*	TAATACGACTCACTATAGGGGGGGACCTGCTCACGGCGG	Mediannikov et al. [87]
CS1258r	*Rickettsia gltA*	ATTAACCCTCACTAAAGATTGCAAAAAGTACAGTGAACA	

### Pathogen screening

DNA samples were screened for *Borrelia burgdorferi* (*s.l*.) and *B. miyamotoi* using qPCR. For *Borrelia burgdorferi* (*s.l*.) we used primers targeting 23S rRNA of *B. burgdorferi* (*s.l*.) (Bb23Sf and Bb23Sr) and a dual-labeled probe, Bb23Sp (Table 1). For *B. miyamotoi* we used primers targeting the *glpQ* gene (Bmi-F and Bmi-R) and a dual-labeled probe, Bmi-P (Table 1). Both assays were carried out in 5 μl reaction volume, including 2.5 μl KAPAProbe FAST PCR Kit, 200 nM forward primer, 200 nM reverse primer, 100 nM probe, 1.25 μl ddH2O, and 1 μl DNA. DNA samples of *B. burgdorferi* (*s.l.*) and *B. miyamotoi* confirmed by sequencing in an earlier study [10] were used as positive controls.

Furthermore, aliquots of original DNA samples were pooled for screening of *Bartonella*, *Rickettsia*, and *Candidatus* Neoehrlichia mikurensis (see Tick collection and sample preparation). For *Bartonella* and *Rickettsia* we used a duplex qPCR assay with primers targeting *Bartonella ssrA* (Bart-ssRA-F and Bart-ssRA-R) and *Rickettsia gltA* (Rspp-F and Rspp-R), and dual-labeled probes Bart-ssRA-P and Rspp-P (Table 1). Assays were carried out in 8 μl reaction volume, including 4 μl KAPAProbe FAST PCR Kit, 200 nM *Bartonella* forward and reverse primers, 300 nM *Rickettsia* forward and reverse primers, 100 nM *Bartonella* probe, 150 nM *Rickettsia* probe, and 3 μl of pooled DNA sample. Patient strains of *Bartonella grahamii* and *B. quintana* were used as positive controls for *Bartonella* assays. Both strains showed successful amplification in every assay. For *Rickettsia* we used a commercially available control sample (ref. MBC042; Vircell, Granada, Spain).

For *Candidatus* Neoehrlichia mikurensis, we used primers targeting the *groEL* gene (CNe-F and CNe-R) and a dual-labeled probe, CNe-P (Table 1). Assays were carried out in 7 μl reaction volume, including 3.5 μl KAPAProbe FAST PCR Kit, 300 nM forward primer, 300 nM reverse primer, 300 nM probe, 0.87 μl ddH2O and 2 μl pooled DNA. No positive controls were available to us for *Ca.* N. mikurensis.

Original, separate DNA samples in pools positive for *Bartonella*, *Rickettsia* or *Candidatus* Neoehrlichia mikurensis were subsequently individually re-analyzed using the qPCR (Table 1). Samples were analysed in 5 μl reaction volume, including 2.5 μl KAPAProbe FAST PCR Kit, 200 nM forward primer, 200 nM reverse primer, 100 nM probe, 1.25 μl ddH2O and 1 μl DNA.

### Sequencing

Samples found positive for *Rickettsia* by qPCR were sequenced using conventional PCR primers (CS877f and CS1258r) targeting *Rickettsia gltA* gene (Table 1). PCR was carried out in 12.5 μl reaction volume containing 3 μl of DNA extract, 2.75 μl ddH2O, 6.25 μl MyTaq Red Mix polymerase mix (product number BIO-25048, Bioline, England), 500 nM forward primer, and 500 nM reverse primer. Water samples were used as blank controls in each PCR batch. Thermal cycling was performed with the following program: 95 °C for 3 min, then 35 cycles of 95 °C for 20 s, 60 °C for 30 s, and 72 °C for 1 min. The PCR products were purified by mixing 1 μl EXO I enzyme, 1 μl rSAP enzyme, 3 μl of ddH2O, and 5 μl of PCR product, after which the samples were first incubated 5 min at 37 °C and then heated 10 min at 80 °C. Purified samples were sent to Macrogen Inc. Europe (The Netherlands) for sequencing. The sequences were trimmed using Geneious version 6 [58] and run through BLAST (www.ncbi.nlm.nih.gov/BLAST/). The trimmed sequences were then further compared to reference sequences of the *Rickettsia* species detected in BLAST, downloaded from GenBank (www.ncbi.nlm.nih.gov/genbank/), to ascertain species using the software Geneious Pro R6 [59]. The reference sequences used in this study were *Rickettsia helvetica* [GenBank: KF447530] and *R. monacensis* [GenBank: KM198341].

## Results

A total of 3169 ticks were collected during the study. All ticks collected by dragging from southwestern Finland (3158 ticks) were identified as *Ixodes ricinus* by qPCR. In addition, eleven samples were collected from a dog in Lempäälä and first identified morphologically [60], and subsequently by means of qPCR, as *I. persulcatus*. Larvae were the most numerous life stage with 1939 individuals collected (61.20 % of all ticks collected), followed by nymphs at 1132 individuals (35.70 %) and adults at 98 individuals (3.10 %). Tick density estimates in each of the study localities are reported in Table 2.

**Table 2:** Tick density estimates and the number of positive samples in each locality in this study

Study locality	Tick density (ticks/100 m^2^)	Tick density (ticks/100 m^2^) without larvae	Tick density in 2000 (ticks/100 m^2^)^d, e^	*B. burgdorferi* (*s.l*.) prevalence^f^		*B. burdorferi* (*s.l*.) prevalence in 2000^d^	
				Samples	Positive (%)	Samples	Positive (%)
Boskär	406.0^g^	36.0	7.3	160	47 (29.4)	272	13 (4.8)
Berghamn	22.0	8.3	0.93	20	1 (5.0)	66	0 (0)
Seili	27.4	5.6	0.28	722	126 (17.5)	69	8 (11.6)
Pähkinäinen	28.3	9.6	0.82	88	18 (20.5)	22	2 (9.1)
Maisaari	2.0	1.6	0.06	18	4 (22.2)	2	0
Vepsä	1.81	1.4	0.2	9	2 (22.2)	8	0
Hirvensalo	2.22	2.0	0.01	9	1 (11.1)	1	0
Ruissalo	0.6	0.6	0.23	10	0	14	0
Samppalinna	0	0	0	0	-	0	-
Urheilupuisto	0	0	0	0	-	0	-
Coastal line^a^	2.0	1.9	n/a	27	4 (14.8)	n/a	
Northern line^b^	0.13	0.13	n/a	3	0	n/a	
Northwestern line^c^	0	0	n/a	0	-	n/a	

^a^Coastal line: Askainen, Kustavi, Lokalahti, Pyhämaa, Pyhäranta

^b^Northern line: Nousiainen, Mynämäki, Laitila, Eurajoki, Luvia

^c^Northwestern line: Alastaro, Matku, Mellilä, Marttila, Paimio

^d^Data from Mäkinen et al. [54]

^e^Tick densities measured for nymphs and adults in 2000

^f^Prevalence measured for nymphs and adults together, as in Mäkinen et al. [54]

^g^In Boskär, larval densities were approximated due to extremely high numbers of larvae (occasionally 500–600 larvae per 50 m drag)

A total of 3169 ticks in 1174 DNA samples were screened for *Borrelia burgdorferi* (*s.l*.), *B. miyamotoi*, *Bartonella*, *Rickettsia,* and *Candidatus* Neoehrlichia mikurensis. DNA samples consisted of 98 adults, 979 nymphs, 60 larval pools (1–111 individuals per sample; 1939 larvae in total), and 37 nymph pools (2–16 individuals per sample; 153 nymphs in total). Individuals from nymph and larval pools were not used in prevalence calculations.

*Borrelia burgdorferi* (*s.l*.) were detected in 217 DNA samples (Table 3). Positive samples consisted of 23 adult ticks, 181 nymphs, and 13 nymph pools (87 individuals). Prevalence of *B. burgdorferi* (*s.l*.) was 23.5 % for adults and 18.5 % for nymphs. The minimum infection rate (MIR) calculated for pooled nymphs was 8.5 %.

**Table 3:** Prevalence of *B. burgdorferi* (*s.l*.), *B. miyamotoi* and *Rickettsia* spp. in single-stored adult and nymphal ticks

Study area	No. of DNA samples			No. (%) of samples positive for *B. burgdorferi* (*s.l*.)			No. (%) of samples positive for *B. miyamotoi*			No. (%) of samples positive for *Rickettsia* spp.		
	Ad^a^	N^a^	Overall	Ad	N	Overall	Ad	N	Overall	Ad	N	Overall
Boskär	11	149	160	1 (9.1)	46 (30.9)	47 (29.4)	0	1 (0.67)	1 (0.63)	0	1 (0.67)	1 (0.63)
Berghamn	1	19	20	0	1 (5.3)	1 (5.0)	0	0	0	0	0	0
Seili	57	665	722	14 (24.6)	112 (16.8)	126 (17.5)	1 (1.8)	3 (0.45)	4 (0.56)	3 (5.3)	10 (1.5)	13 (1.8)
Pähkinäinen	5	83	88	2 (40.0)	16 (19.3)	18 (20.5)	0	1 (1.2)	1 (1.1)	1 (20.0)	0	1 (1.1)
Maisaari	2	16	18	1 (50.0)	3 (18.8)	4 (22.2)	0	0	0	0	0	0
Vepsä	4	5	9	2 (50.0)	0	2 (22.2)	0	0	0	0	0	0
Ruissalo	3	7	10	0	0	0	0	0	0	1 (33.3)	0	1 (10.0)
Hirvensalo	0	9	9	-	1 (11.1)	1 (11.1)	-	0	0	-	0	0
Coastal line	4	23	27	2 (50.0)	2 (8.7)	4 (14.8)	0	0	0	0	0	0
Northern line	0	3	3	-	0	0	-	0	0	-	0	0
Lempäälä	11	0	11	1 (9.1)	-	1 (9.1)	0	-	0	0	-	0
Total	98	979	1077	23 (23.5)	181 (18.5)	204 (18.9)	1 (1.02)	5 (0.51)	6 (0.56)	5 (5.1)	11 (1.1)	16 (1.5)

^a^ *Abbreviations*: Ad adults; N nymphs

*Borrelia miyamotoi* were detected in 16 DNA samples (Table 3). These consisted of one adult male, five individual nymphs, four nymph pools (21 individuals) and six larval pools (282 individuals). *B. miyamotoi* prevalence was 1.02 % for adults and 0.51 % for individual nymphs. Minimum infection rates (MIR) were 2.61 % for pooled nymphs and 0.31 % for pooled larvae.

*Rickettsia* spp. were detected in 21 DNA samples (Table 2). Positive samples consisted of five adults, 11 nymphs, one nymph pool (two individuals), and four larval pools (216 individuals). These accounted for a prevalence of 5.10 % for adults and 1.10 % for nymphs. Minimum infection rates were 0.65 % for pooled nymphs and 0.21 % for pooled larvae. Furthermore, 20 out of the 21 positive samples were sequenced to determine *Rickettsia* spp. One sample had ran out prior to this and could therefore not be sequenced. Sixteen sequenced samples were identified as *R. helvetica* and three as *R. monacensis* (sequence identity 99–100 % with corresponding reference sequences)*.* One sample could not be identified due to poor quality of sequencing data.

Site-specific prevalence of the detected pathogens is reported in Table 3. Regarding pooled samples, which are not reported in Table 3, *B. miyamotoi* were found from two nymph pools from Seili and six larval pools from Boskär. *Rickettsia* spp. were found from a single nymph pool and three larval pools from Seili and one larval pool from Boskär.

Co-infection by *B. burgdorferi* (*s.l*.) and *Rickettsia* was detected in three samples. These samples were an adult male and two nymphs from Seili. All co-infecting *Rickettsia* spp. were identified as *R. helvetica*. The rate of double infections was 1.02 % for adults and 0.20 % for nymphs. Both *B. miyamotoi* and *R. monacensis* were detected in a larval pool from Boskär, but whether they were carried by the same individual could not be determined.

All samples were negative for *Bartonella* spp. and *Candidatus* Neoehrlichia mikurensis.

## Discussion

In the present study, ticks were collected by cloth dragging from 25 localities around southwestern Finland. The highest densities of *I. ricinus* were measured from rural islands in the Turku Archipelago, which were also previously studied in 2000 [54] (Fig. 1, Table 2). Compared to previous results, higher tick densities were observed in all study localities that yielded ticks. However, these temporal comparisons were made between two years only, except in Seili, where high tick densities were measured also in 2012 and 2015 (Sormunen *et al.* [10] and unpublished). Further surveys of tick abundance in the study localities are required to more conclusively determine if an increase in tick abundance has occurred and whether similar tick densities can be observed persistently.

A decrease in tick densities was observed when moving to the mainland from the archipelago and/or inland from the coastline (Table 2, Fig. 1). No ticks were found from the two city parks in Turku (Samppalinna and Urheilupuisto) in either 2000 or 2013 [54], despite similar localities in the capital city Helsinki having high tick densities (mean 16.3/100 m^2^) [56]. It should be noted, however, that our studies in the city parks were focused mainly on assessing the threat that ticks pose to humans. Therefore, dragging was conducted mostly in locations utilized by citizens for recreational purposes, which were not necessarily optimal for ticks. Nevertheless, some ticks would be expected to have been caught if densities were similar to those observed in Helsinki. Thus, it seems that some unidentified ecological factors (such as certain important host animals) affecting *Ixodes* spp. occurrence are still lacking in the city parks of Turku, as suggested previously by Mäkinen and colleagues [54].

*Borrelia burgdorferi* (*s.l*.) was the most prevalent of the screened pathogens. Similar results have been reported in other studies concerning the prevalence of various pathogens found from *I. ricinus* in Europe [30, 47, 61–63]. Furthermore, the life stage distribution of infected ticks conforms to results reported from all over Europe [64, 65], with adults having noticeably higher *B. burgdorferi* (*s.l*.) prevalence than nymphs. The adult and nymphal prevalence observed in the current study (23.5 and 18.5 %) were somewhat higher than the averages reported from Europe (18.6 and 10.1 %, respectively) [64], though much geographical variation in prevalence is known to exist. All larval samples analyzed were negative for *B. burgdorferi* (*s.l*.) infection. Since prevalence was high in adults and nymphs collected from the same study localities, this finding supports the notion that transovarial transmission of *B. burgdorferi* (*s.l*.) from adult females to larvae is at most an exceedingly rare occurrence [66].

Mäkinen and colleagues previously investigated *B. burgdorferi* (*s.l*.) prevalence of *I. ricinus* in ten of the study localities investigated in the present study [54]. Compared to these results from the year 2000, an apparent increase in prevalence can be observed across all study localities that yielded ticks, apart from Ruissalo (Table 2). While the results of the comparison presented here should be interpreted with caution due to relatively low sample sizes, the uniformity of observed changes suggests that an actual increase in prevalence has occurred in at least some of the study localities (e.g. Boskär, Seili, and Pähkinäinen).

*Borrelia miyamotoi* was found from all tick life stages (infection rates for adults: 1.02 %; individual nymphs 0.51 %; pooled nymphs (MIR) 2.61 %; and pooled larvae (MIR) 0.31 %). The prevalence observed for adults and nymphs in the current study conforms to surveys conducted elsewhere in Europe, which have reported modest prevalence rates (0.17–3.8 %) [8, 67, 68]. In Finland, screening of individual ticks for *B. miyamotoi* has previously been conducted only on the Åland Islands [9]. As a results, only this single reference for typical *B. miyamotoi* prevalence exists (0.51 % overall; 0.81 % for adults; 0.20 % for nymphs) [9]. Because higher prevalences have been observed elsewhere [69–71], it is evident that *B. miyamotoi* spirochetes can achieve higher prevalence given suitable conditions and time. Therefore, foci with low prevalence could be either recently infested or in ecologically suboptimal areas. In samples from Seili island, *B. miyamotoi* has been identified for three consecutive years (2012–2014; Table 3; see Sormunen et al. [10] for 2012 data), though prevalence has remained low (0.47–0.68 %). These consecutive findings suggest that Seili is a locality endemic for the spirochete. *Borrelia miyamotoi* has now been reported from four localities in Finland: the Åland Islands and Seili Island previously [9, 10], and Pähkinäinen and Boskär in the current study (Table 3).

*Rickettsia* spp. were found from 5.10 % of adult and 1.10 % of nymphal ticks (overall prevalence 1.50 %). There seems to be great variation in *Rickettsia* spp. prevalence in European tick populations, ranging from values similar to those reported here to considerably higher (1.9–58 % for adults; 1.12–18 % for nymphs; 1.7–16 % in studies where adults and nymphs were combined) [26, 28, 62, 63, 72–74]. While *Rickettsia* spp. have been reported from ticks in many European countries, they have not been screened from Finnish ticks before. In neighboring Sweden, with similar climatic conditions, high variation in *Rickettsia* spp. prevalence has been observed [26–29]. Most *Rickettsia* spp. reported from Swedish ticks have been identified as *R. helvetica*. In the current study, *R. helvetica* and *R. monacensis* were detected in ticks collected from southwestern Finland (Table 3). While the presence of *R. helvetica* was expected based on the studies conducted in Sweden, the detection of *R. monacensis* was somewhat unexpected. This species has been reported from ticks in nearby Poland and Estonia [32, 75], but no findings have been reported from questing ticks in the northern countries of the Baltic region. The ecology and epidemiology of *R. monacensis* are still relatively unknown, but patient cases from Spain and Italy suggests that it is capable of human infection [35, 76]. With the presence of at least two potentially pathogenic species confirmed in ticks in south-western Finland, further efforts should be made to more broadly study the occurrence and prevalence of *Rickettsia* in Finnish ticks.

As initial pathogen uptake and infection of ticks usually takes place during feeding, the role of *I. ricinus* and *I. persulcatus* larvae as vectors for human infections is mostly limited to pathogens capable of transovarial transmission. Consequently, larvae seem to have received less attention than adult and nymphal ticks in studies of tick-borne diseases [64]. However, recent studies have revealed that *B. miyamotoi*, *Ca.* N. mikurensis, and certain species of *Rickettsia* are likely capable of both transovarial transmission and human infection [11–15, 19, 66, 67, 77–79]. In the present study, *R. helvetica*, *R. monacensis*, and *B. miyamotoi* were detected in larval samples. Minimum infection rates for pooled larvae were 0.21 % for *Rickettsia* spp. and 0.31 % for *B. miyamotoi*. These findings suggest that these bacteria can indeed be found from wild questing *I. ricinus* larvae, thus supporting the notion that transovarial transmission occurs naturally in tick populations. Consequently, tick larvae should also be considered as potential health risks in areas endemic for *B. miyamotoi* and *Rickettsia* spp. 

All screened samples of this study were negative for *Bartonella* and *Ca.* N. mikurensis infection. However, no positive controls for *Ca.* N. mikurensis were available to us at the time of the analysis. Therefore, the negative results regarding *Ca.* N. mikurensis should be interpreted with care. These pathogens have not been screened from Finnish ticks before. *Candidatus* N. mikurensis has been reported from ticks and/or host animals in nearby Germany, Russia, Sweden, and Denmark [47–52]. *Bartonella* have been reported from ticks in Germany and Russia, but surveys in Sweden have been negative [37, 38, 80]. While the relevance of ticks as vectors for human bartonellosis is still debated [36, 81, 82], *I. ricinus* has been shown to be a competent vector for some *Bartonella* spp. [39, 43]. Still, as far as we are aware, no patient cases of human bartonellosis have been incontrovertibly linked to tick bites. The same is true for *Ca.* N. mikurensis patient cases reported from Sweden, Switzerland, Germany and the Czech Republic in Europe [44–46]. However, for *Ca.* N. mikurensis, knowledge regarding other possible vectors for human infection is missing. As the prevalence and ability of these pathogens to be transmitted to humans via a tick bite are still under survey, it is hard to evaluate the impact they can potentially have on national welfare. Nevertheless, further screening should be conducted to determine more conclusively if they occur in Finnish ticks.

## Conclusions

In the present study we reported for the first time the occurrence and prevalence of *Rickettsia* spp. in Finnish ticks. Furthermore, the presence of *Bartonella* spp. and *Candidatus* Neoehrlichia mikurensis in Finnish ticks was screened for the first time. Despite the absence of *Bartonella* spp. and *Ca.* N. mikurensis in screened samples, further studies should be conducted regarding their occurrence and prevalence in Finland. *Borrelia* spp. prevalence of ticks collected from south-western Finland was 19.5 %, and *B. miyamotoi* was reported from two new localities in the Archipelago Sea. *Rickettsia* spp. and *B. miyamotoi* were also found from larval pools, supporting the notion of naturally occurring transovarial transmission of these pathogens, and emphasizing the importance larvae may have as vectors for human infections. The epidemiological significance of the detected *Rickettsia* spp. and *B. miyamotoi* is still unclear and should be further assessed. Finally, with indications of an increase in tick abundance and tick-borne pathogen diversity in Finland, a wider and more comprehensive survey regarding tick-borne pathogens should be made a priority.
